# Cognición y COVID Persistente: Una Revisión Sistemática PRISMA de Estudios Longitudinales

**DOI:** 10.31083/RN37385

**Published:** 2025-01-24

**Authors:** María Alejandra Tudorache Pantazi, Marien Gadea-Doménech, Raúl Espert Tortajada

**Affiliations:** ^1^Unidad de Neuropsicología (Servicio de Neurología), Hospital Clínico Universitario de València, 46010 Valencia, España; ^2^Departamento de Psicobiología, Facultad de Psicología, Universitat de València, 46010 València, España

**Keywords:** *Long* COVID, cognición, COVID-19, SARS-CoV-2, estudios longitudinales, revisión sistemática, Long COVID, cognition, COVID-19, SARS-CoV-2, longitudinal studies, systematic review

## Abstract

**Introducción:**

El *Long *COVID o (Covid persistente) es definido por el National Institute for Health and Care Excellence (NICE) como el conjunto de signos y síntomas que se desarrollan durante o después de una infección por SARS-CoV-2 y que continúan durante más de doce semanas sin ningún tipo de diagnóstico alternativo. Uno de los síntomas persistentes más frecuentes reportados por los pacientes y comprobados en estudios de neuroimagen es la disfunción cognitiva, debida a una hipoconectividad generalizada y una lesión axonal difusa en sustancia blanca. Es por ello por lo que los objetivos de la presente revisión son determinar el tiempo que permanecen afectadas las funciones cognitivas durante el *Long* COVID y explorar cuáles son las funciones cognitivas que se encuentran más afectadas más allá de los tres meses de seguimiento en pacientes de hasta 65 años sin complicaciones neuropsicológicas o psiquiátricas previas.

**Métodos:**

Se realizó una revisión sistemática con criterios PRISMA y se incluyeron 11 artículos mediante una búsqueda exhaustiva en cinco bases de datos diferentes: PubMed, Medline, Scopus, WOS y ProQuest. El riesgo de sesgo de los artículos se evaluó mediante la escala Newcastle-Ottawa.

**Resultados:**

Los problemas cognitivos en el *Long *COVID persisten a lo largo del tiempo y mejoran lentamente, aunque los estudios parecen coincidir en que la mayoría de las áreas mejoraron significativamente a partir del año. Las funciones cognitivas que más tiempo permanecieron afectadas fueron la velocidad de procesamiento y la atención.

**Conclusiones:**

Estas alteraciones cognitivas provocan una reducción en la calidad de la vida y capacidad de trabajo de los pacientes y manifiestan la necesidad de una intervención cognitiva.

## Abstract (en)

**Introduction:**

*Long* COVID is defined by National Institute for Health and Care Excellence (NICE) as the set of signs and symptoms that develop during or after a SARS-CoV-2 infection and continue for more than twelve weeks without any alternative diagnosis. One of the most frequent persistent symptoms reported by patients and verified in neuroimaging studies is cognitive dysfunction, due to a generalized hypoconnectivity and a diffuse axonal lesion in white matter. Therefore, the objectives of the present review are to determine how long cognitive functions remain affected during Long COVID and to explore which cognitive functions are most affected beyond three months of follow-up in patients up to 65 years of age without previous neuropsychological or psychiatric complications.

**Methods:**

A systematic review was performed using PRISMA criteria and 11 articles were included through a comprehensive search of five different databases: PubMed, Medline, Scopus, WOS and ProQuest. The risk of bias of the articles was assessed using the Newcastle-Ottawa scale.

**Results:**

Cognitive problems in *Long* COVID persist over time and improve slowly, although studies seem to agree that most areas improved significantly after one year. The cognitive functions that remained impaired the longest were processing speed and attention.

**Conclusions:**

These cognitive alterations cause a reduction in the quality of life of the patients and a reduction in work capacity and manifest the need for a cognitive intervention.

## 1. Introducción

El COVID-19 es la enfermedad causada por el agente infeccioso SARS-CoV-2 que, desde la pandemia del 2020, ha dejado más de 760 millones de casos en todo el mundo con más de 6,9 millones de fallecimientos [1]. Pese a ser una enfermedad conocida por afectar, fundamentalmente, al tracto respiratorio superior, se ha demostrado a lo largo de estos años que se trata de una patología que presenta una afectación multisistémica muy variada [2]. Se estima que entre un 10 y un 15% de los pacientes infectados pueden llegar a desarrollar una fase de la enfermedad llamada *Long* COVID [3] y una estimación reciente apunta a un número de más de 65 millones de personas con esta condición [4, 5]. El Long COVID (o también conocido como COVID persistente) es definido por el National Institute for Health and Care Excellence (NICE) [6] como el conjunto de signos y síntomas que se desarrollan durante o después de una infección por SARS-CoV-2 y que continúan durante más de doce semanas sin ningún tipo de diagnóstico alternativo que pueda explicar esos síntomas. Esta condición de la enfermedad ha recibido otras denominaciones en la literatura como COVID residual o a *Post-Acute Sequelae SARS-CoV-2 *(PASC), para referirse a las secuelas de la enfermedad.

Uno de los síntomas más frecuentes descritos durante el *Long* COVID es la disfunción cognitiva [7, 8, 9, 10, 11]. Esta provoca una reducción en la calidad de vida y la capacidad de trabajo [12, 13] y se ha establecido como uno de los motivos más habituales de baja laboral tras la pandemia, junto con los síntomas afectivos. Los mecanismos de neuroinvasión y neuroinflamación generalizada del SARS-CoV-2, proporcionan información acerca de por qué los síntomas cognitivos figuran entre los más informados por los Neurólogos y Neuropsicólogos. En muchos pacientes el virus consigue llegar al cerebro a través de la placa cribiforme hasta el bulbo olfatorio, o directamente por la circulación sanguínea atravesando la Barrera Hematoencefálica (BHE) [14]. Debido a esta neuroinvasión, algunas de las áreas más afectadas son la sustancia gris, el giro parahipocampal y el córtex orbitofrontal. Dichas estructuras son las que se encuentran relacionadas con el correcto desempeño de la memoria, las funciones ejecutivas y emocionales [15].

La literatura científica no ha parado de crecer en relación con la búsqueda de factores de riesgo que puedan prever qué pacientes pueden ser más vulnerables a desarrollar *Long* COVID tras la infección. Algunas investigaciones han encontrado que determinados síntomas durante la fase aguda como la disgeusia, la anosmia o la cefalea se han relacionado con un peor rendimiento en pruebas neuropsicológicas [15, 16, 17, 18]. Asimismo, también se ha visto que aquellas personas con problemas de inmunidad presentarían más síntomas cognitivos, y que el 50% de los casos de *Long* COVID se dan entre edades comprendidas entre los 36 y 50 años, y el 80% de los casos son reportados por mujeres [12]. En la misma línea, se ha encontrado un perfil educativo más alto como uno de los pocos factores protectores investigados que dificultan la detección de déficits cognitivos [13, 19]. La mejora de la microbiota intestinal -que se ve alterada por la infección del SARS-CoV-2– parece estar también relacionada con la ausencia de desarrollo de síntomas persistentes característicos del *Long *COVID [20], aunque todavía es un tema que se encuentra en vías de investigación.

Por otra parte, con respecto a las funciones cognitivas más afectadas, la mayoría de los estudios que realizaron un seguimiento de 3 meses constataron una mayor afectación en la velocidad de procesamiento, las funciones ejecutivas y la memoria [15, 21, 22]. Sin embargo, todavía quedan muchas incógnitas por esclarecer respecto al impacto persistente del COVID-19 en las funciones cognitivas y muchos artículos manifiestan la necesidad de investigaciones longitudinales que capten la evolución de estos síntomas. Es por ello por lo que la presente revisión sistemática pretende recopilar todos los estudios longitudinales disponibles hasta el momento para resolver las dos siguientes preguntas de investigación: En primer lugar: ¿Cuánto tiempo permanecen afectadas las funciones cognitivas durante el *Long* COVID? En segundo lugar: ¿Cuáles son las funciones cognitivas que se encuentran más afectadas más allá de los tres meses de seguimiento?

## 2. Pacientes y Métodos

Se realizó una revisión sistemática de estudios longitudinales con más de tres meses de seguimiento de las funciones cognitivas utilizando los criterios PRISMA (the checklist is in **Supplementary Material-PRISMA_2020_checklist**) [23]. En primer lugar, para la selección de artículos se aplicaron las directrices PICO (P: Participantes con una edad comprendida entre 18 y 65 años diagnosticados mediante una reacción en cadena de la polimerasa (PCR) y sin un historial de complicaciones neurológicas o de trastornos psiquiátricos previos; I: Estudios que empleen baterías neuropsicológicas estandarizadas con propiedades psicométricas adecuadas y baremos adecuados a la población pasadas en un primer momento para realizar una línea base; C: Baremos adecuados a la población o grupo control diagnosticado en las mismas condiciones que el experimental y sin deterioro cognitivo previo; O: Resultados de artículos que incluyan como una de las variables centrales la cognición y que preferiblemente controlen variables emocionales).

### 2.1 Búsqueda Inicial 

Las primeras búsquedas se realizaron desde diciembre de 2023 hasta febrero de 2024 en las bases de datos de PubMed, Medline y Web of Science (WOS). Se introdujeron los términos ‘COVID-19’, ‘SARS-CoV-2’, ‘*cognition*’, ‘*neuropsychology*’ y ‘Neurocovid-19’ usando los operadores booleanos *AND* y *OR* según conveniencia. Estas búsquedas permitieron revisar la literatura científica disponible hasta el momento acerca del COVID-19 y la cognición y formular las dos preguntas de investigación que permitiesen el avance de la cuestión.

### 2.2 Búsqueda Sistemática 

La búsqueda sistemática abarcó desde febrero de 2024 hasta el 20 de agosto de 2024 y se ampliaron las bases de datos a Scopus y ProQuest para facilitar la captación de la totalidad de artículos disponibles hasta el momento. En todas se utilizaron los términos ‘COVID-19’, ‘SARS-CoV-2’, ‘*Long *COVID’, ‘*neuropsycholoy*’, ‘*cognition*’, ‘*cognitive impairment’*, ‘*longitudinal studies*’ y la misma combinación de los operadores booleanos *AND* y *OR. *La combinación de términos que arrojó mejores resultados fue ‘*Longitudinal studies*’ *AND* ‘COVID-19’ *AND* ‘*cognition*’. Los criterios de búsqueda utilizados permitieron rastrear artículos que estuviesen disponibles con el texto completo gratuito en inglés y en español, y que datasen de los últimos cinco años. Antes de proceder a la selección de artículos, se definieron los criterios de inclusión y exclusión.

#### 2.2.1 Criterios de Inclusión

– Estudios que cumplan las directrices PICO mencionadas anteriormente.

– Estudios longitudinales.

– Muestras de tamaño considerables (n ≥ 50).

– Artículos correspondientes al rango temporal 2020–2024, publicados en inglés y en español y de libre acceso.

#### 2.2.2 Criterios de Exclusión

– Otras revisiones sistemáticas, metaanálisis, estudios de caso o estudios con animales.

– Estudios transversales.

– Artículos no disponibles gratuitamente (Open Access) o pertenecientes a revistas no científicas o no indexadas en el Journal of Citation Reports (JCR).

– Estudios que cuenten con medidas de autoinforme para medir la cognición.

– Estudio con pruebas neuropsicológicas pasadas exclusivamente de manera telefónica.

### 2.3 Proceso de Selección y de Extracción de Datos

**Fig. 1.**
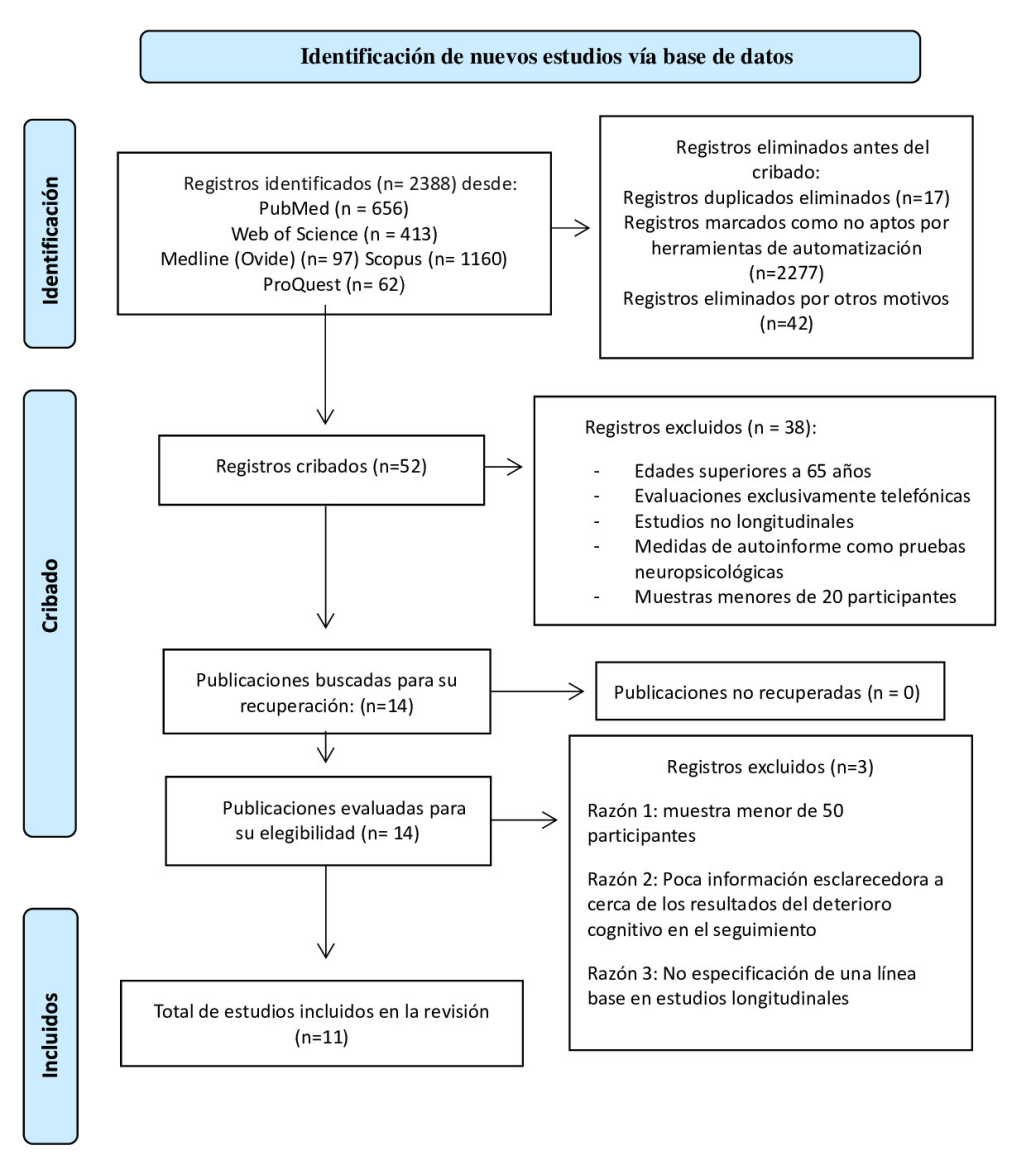
**Diagrama de flujo PRISMA con tres niveles, de acuerdo con los criterios de [23]**.

Tras la lectura del título junto con el *abstract* se consideraron adecuados 52 artículos que previanmente se organizaron mediante el gestor de referencias Mendeley que ayudó a eliminar los duplicados. Tras aplicar los criterios de inclusión y exclusión, junto con las directrices PICO, finalmente, se seleccionaron 11 artículos. Se trató de una revisión por pares con un porcentaje de concordancia del 95% entre los dos autores principales (M. A. T. P. y R. E. T). Este proceso se puede consultar en la Fig. 1 (Ref. [23]). Posteriormente, se extrajeron y se sistematizaron en tablas datos como los siguientes: autor, año de publicación, diseño del estudio, duración de la evaluación y seguimiento, tamaño muestral, características del grupo experimental, pruebas neuropsicológicas y principales diferencias significativas encontradas (Tabla 1, Ref. [24, 25, 26, 27, 28, 29, 30, 31, 32, 33, 34]).

**Table 1.** **Resumen de las características de los estudios incluidos**.

Estudio	Tipo	Evaluación y Seguimiento	Muestra	Edad Media Grupos	Pruebas Neuropsicológicas	Diferencias significativas
Martin *et al*. (2024) [24]	Longitudinal con grupo control sin COVID	L.B. al año y 6 meses de seguimiento	N = 88 (76,1% M) L.B. n = 50 GC (80% M) n = 77 6 meses	Grupo COVID: 46,67 años (21–64) Grupo control: 45,98 años (24–65)	Test of attentional performance (TAP): estado de alerta tónico y fásico, atención dividida, inhibición; Screening module of the Neuropsychological Assessment Battery Screening (S-NAB): atención, funciones ejecutivas, lenguaje, percepción, memoria verbal y visual.	Diferencias estadísticamente significativas en medidas de menor velocidad de procesamiento en pacientes con PCS frente a los controles en la L.B. Los PCS siguieron presentando un perfil de déficit persistente en la velocidad de procesamiento a los 6 meses salvo en lenguaje y memoria.
Del Corral *et al*. (2024) [33]	Longitudinal sin grupo control en pacientes no hospitalizados	L.B. a los 3 meses y a 6–7 meses seguimiento	N = 102 (38 H y 64 M)	Grupo pacientes: 46,6 años (±14.1 SD)	Evaluación cognitiva de Montreal (MoCA): atención, concentración, funciones ejecutivas, memoria, lenguaje, habilidades visoconstructivas y visuoespaciales, pensamiento conceptual, cálculo y orientación temporal y espacial.	El 55,9% experimentó deterioro cognitivo, aunque hubo una mejora estadísticamente significativa a los 6–7 meses.
Vasile *et al*. (2023) [29]	Observacional prospectivo de formas moderadas y graves de COVID sin GC	L.B, 6 meses y 1 año	N = 137 (56% M) n = 66 (18–44 años) n = 71 (45–60 años)	Grupo pacientes: Entre 18–60 años	Evaluación cognitiva de Montreal (MoCA) y Mini-Mental State Examination (MMSE): orientación espacial y temporal, atención y concentración, memoria a corto plazo, capacidad visuoespacial y capacidad de comprender y seguir instrucciones.	A los 6 meses el 95% de los pacientes obtuvieron puntuaciones normales en ambas escalas, sin embargo, las funciones cognitivas con más afectación y a lo largo del tiempo fueron la atención, la memoria de trabajo y las funciones ejecutivas.
Poletti *et al*. (2022) [30]	Longitudinal con grupo control	6 meses	N = 312 (n = 92 mes 1; n = 122 mes 3; n = 98 mes 6 y n = 60 mes 3 y 6) GC con TDM: 165 y GC = 165	Grupo COVID: 49,61 años (±8,81 DE) GC con TDM: 49,41 años (±11,19 DE) Grupo control: 40,57 años (±11,79 DE)	Evaluación Breve de la Cognición en la Esquizofrenia (BACS): memoria verbal, fluidez verbal, memoria de trabajo, atención selectiva, velocidad de procesamiento, coordinación psicomotora y funciones ejecutivas.	El 75% de los pacientes a los 3 y 6 meses mostraron deterioro cognitivo en al menos una función ejecutiva. Las funciones cognitivas más afectadas respecto al grupo control fueron la atención, la velocidad de procesamiento y la coordinación motora.
Stavem *et al*. (2022) [34]	Longitudinal multicéntrico sin grupo control en pacientes no hospitalizados	8 y 13 meses después	N = 305 L.B. (n = 234 evaluación cognitiva seguimiento y 59% M)	Grupo COVID: 49,8 años (±14,7 DE)	Batería automatizada de pruebas neuropsicológicas de Cambridge (CANTAB): memoria a corto plazo, atención y función ejecutiva.	Los resultados en la muestra de pacientes no hospitalizados infectados por COVID-19 entre 8 y 13 meses después de la fase aguda fueron solo marginalmente peor a lo esperado en comparación con los datos normativos de Reino Unido en memoria a corto plazo, procesamiento visuoespacial, aprendizaje y atención.
Steinmetz *et al*. (2023) [28]	Estudio de cohorte prospectivo longitudinal sin grupo control	L. B a los 8 meses en promedio y seguimiento de 3 y 6 meses	L. B. (T_0_) = 158 A los 3 meses (T_1_): N = 134 A los 6 meses (T_2_): N = 104	Grupo COVID N = 158 (48,2 años ±14,3 DE) n = 124 M (48,5 años ±13,8 DE) n = 34 H (47,1 años ±16,1 DE)	Montreal Cognitive Assessment (MoCA).	Mejorías en el MoCA a los 3 meses y mejorías todavía aún más significativas a los 6 meses, sin embargo, el 25% todavía presenta puntuaciones anormales a los 6 meses frente al 38,9% de la línea base.
Lynch *et al*. (2024) [27]	Longitudinal sin grupo control	L.B a los 7 meses y 6 meses de seguimiento	N = 75 (70,7% M) n = 63 6 meses	Grupo COVID: 43,5 años (±15)	RBANS Forma A and B. TMT Forma A and B.	El 51% sigue teniendo un rendimiento bajo o extremadamente bajo en las pruebas con puntuaciones estadísticamente más bajas en lenguaje y mejora sobre todo en memoria demorada.
Muschel *et al*. (2023) [25]	Longitudinal sin grupo control	A los 7 meses	N = 74 (70% M) (n = 51 NCC y n = 23 CC)	Grupo Total: 43,49 años (±15,06 DE) Grupo NCC: 40,39 años (±14,77 DE) Grupo CC: 50,35 años (±13,61 años)	Test of Premorbid Functioning (TOPF): atención, memoria verbal y auditiva y visual inmediata y retardada, habilidades visuoespaciales y constructivas, velocidad psicomotora, función ejecutiva, RBANS Form A, TMT Parts A and B, Letter Fluency, Stroop Test, MoCA.	Puntuaciones estadísticamente significativas por debajo de las normas poblacionales esperadas en atención, concentración, memoria remota y funciones ejecutivas.
Allam *et al*. (2023) [26]	Estudio observacional longitudinal	Al mes, 3 y 6 meses	N= 50 (64% M)	Grupo COVID: 35,4 años (±10,6)	WMS-R: Memoria estímulos verbales y figurativos, material abstracto y significativo, recuerdo retardado e inmediato. WCST: Funciones ejecutivas.	A pesar del impacto negativo del COVID-19 en la cognición los participantes mejoraron gradualmente con el tiempo, a excepción de la memoria visual.
Shanley *et al*. (2022) [32]	Estudio de cohorte prospectivo longitudinal	L. B. a las 15,6 semanas (media) y seguimiento de 6 meses	L.B. N = 56 (n = 40 SAN, n = 16 CAN; n = 44 L.B y n = 19 a 6 meses 78% M)	Grupo total: 50 años (±13,6 DE) Grupo SAN: 50,5 años (IQR = 26,25) Grupo CAN: 48,8 años (IQR = 15,5)	Montreal Cognitive Assessment (MoCA): funcionamiento cognitivo, abstracción, MCP, lenguaje, orientación, habilidades visuoespaciales. Funciones intelectuales: TOPF, WASI-2. Atención y memoria de trabajo: WAIS-IV, PASAT. Velocidad de procesamiento: TMT Part A, SDMT. Funciones ejecutivas: Stroop Test, TMT Part B. Lenguaje: COWAT, Animal Fluency, BNT. Memoria: CVLT-II, BVMT-R.	El 75% del grupo sin afecciones neurológicas mejoraron o no obtuvieron ningún cambio en sus puntuaciones y el 25% presentó un empeoramiento en el MoCA con fallos frecuentes en recuerdo demorado, lenguaje y atención.
Almeria *et al*. (2024) [31]	Longitudinal sin grupo control	L.B. a los 10–30 días y seguimiento de 6 meses	L.B. N = 200 (n = 108 a los 6 meses) (59,25% H)	Grupo COVID: 49,10 años (±7,67)	Test de Aprendizaje Verbal España Complutense (TAVEC), reproducción visual de la Wechsler Memory Scale IV (WMS-IV), Dígitos directos e inversos, Letras y números, Trail Making Test (TMT A and B), SDMT, Test de Stroop, Fluencia fonética y semántica y Test de Denomnación de Boston (BNT) del proyecto NEURONORMA.	En el seguimiento a los 6 meses se observó una mejoría global significativa en las subpruebas de memoria verbal y visual, velocidad de procesamiento, función ejecutiva y denominación, independientemente de la gravedad de la enfermedad y las quejas cognitivas.

L.B, Línea Base; M, Mujeres; H, Hombres; PCS, Síndrome Post-COVID; GC, Grupo control; TDM, Trastorno Depresivo Mayor; DE, Desviaciones estándar; MCP, Memoria a corto plazo; IQR, Rango intercuartil; T, Tiempo; NCC, *No cognitive Complaints* (sin quejas cognitivas); CC, *Cognitive Complaints *(con quejas cognitivas); SAN, Sin Afecciones Neurológicas; CAN, Con Afecciones Neurológicas; TOPF, Test of Premorbid Functioning; RBANS, Repeatable Battery for the Assessment of Neuropsychological Status; TMT, Trail Making Test; WMS-R, Wechsler Memory Scale Revised; WCST, Wisconsin Card Sort; WASI-2, Wechsler Abbreviated Scale of Intelligence-2nd Edition; WAIS-IV, Wechsler Adult Intelligence Scale-4th Edition; PASAT, Paced Auditory Serial Addition Test; SDMT, Symbol Digit Modality Test; COWAT, Controlled Oral Word Association Test; BNT, Boston Naming Test; CVLT-II, California Verbal Learning Test 2nd Edition; BVMT-R, Brief Visuospatial Memory Test-Revised; SAN, sin afecciones neurológicas.

### 2.4 Evaluación de la Calidad Metodológica

El riesgo de sesgo de los 11 artículos se evaluó mediante la escala de Newcastle-Ottawa [35]. Un instrumento muy útil para estudios donde no fue posible realizar una asignación aleatoria. Está basado en un sistema de estrellas para otorgar puntuaciones y permitió evaluar cómo fue realizada la selección de la muestra, si se contó con un grupo control adecuado, si se comprobó la exposición realizando las PCR correspondientes o si hubo mucha pérdida de la muestra. Las puntuaciones que se derivan de aplicar la escala permitieron comprobar si los artículos contaban con una alta calidad, o si por el contario contaban con un alto o muy alto riesgo de sesgo.

Durante la evaluación de los artículos, se realizaron evaluaciones independientes por los dos autores principales (M. A. T. P. y R. E. T.) y se resolvieron las discrepancias para llegar a un acuerdo común respecto a la calificación de cada artículo.

## 3. Resultados

Los artículos incluidos en esta revisión, en su mayoría, pertenecen a revistas con altos índices de impacto (Q1 y Q2) según el JCR. Sin embargo, encontramos que, casi todos los estudios longitudinales disponibles sobre los problemas cognitivos en el *Long* COVID en población de 18 a 65 años, cuentan con unos niveles de riesgo de sesgo muy altos. Esto se puede observar en la Tabla 2 (Ref. [24, 25, 26, 27, 28, 29, 30, 31, 32, 33, 34, 35]), donde se incluyen las puntuaciones obtenidas de cada artículo en la escala de Newcasttle-Ottawa. Es importante tener en cuenta que la mayoría de los estudios obtienen su muestra de programas de rehabilitación para personas que padecen PASC o pacientes que acuden al hospital con quejas por síntomas persistentes. Esto ya supone un sesgo de selección en la muestra, por lo que en la presente revisión las conclusiones generalizadas con respecto a la gravedad y el curso del deterioro cognitivo en el *Long *COVID resultan limitadas. Así mismo, solamente se encontraron dos estudios [24, 25] que incluyesen un grupo control, por lo que estas condiciones, en conjunto, explicarían las puntuaciones tan bajas obtenidas en esta escala.

**Table 2.** **Análisis del riesgo de sesgo según la Escala de Newcastle-Ottawa [35]**.

Autores y año	Categorías	S	S	S	S	C	C	E	E	E	Puntuación Total
	Diseño del estudio	1	2	3	4	1a	1b	1	2	3	
Martin *et al*. (2024) [24]	Casos y controles	★	★		★	★					4
Del Corral *et al*. (2024) [33]	Casos y controles	★							★	★	3
Stavem *et al*. (2022) [34]	Casos y controles	★						★			2
Muschel *et al*. (2023) [25]	Casos y controles	★						★		★	3
Shanley *et al*. (2022) [32]	Casos y controles	★						★			2
Allam *et al*. (2023) [26]	Casos y controles	★									1
Lynch *et al*. (2024) [27]	Casos y controles	★						★			2
Steinmetz *et al*. (2023) [28]	Casos y controles							★			1
Poletti *et al*. (2022) [30]	Casos y controles	★		★	★			★			4
Vasile *et al*. (2023) [29]	Casos y controles	★						★			2
Almeria *et al*. (2024) [31]	Casos y controles	★	★					★		★	4

★: Símbolo de puntuación de la escala. Los números corresponden a los siguientes ítems de la Escala de Newcastle-Ottawa: SELECCIÓN (S): 1. ¿Es adecuada la definición de caso?; 2. Representatividad de los casos; 3. Selección de controles; 4. Definición de control. COMPARABILIDAD (C): 1. Comparabilidad casos y controles; 1a. Para el factor más importante; 1b. Para otros factores de la investigación. EXPOSICIÓN (E): 1. Exposición verificada; 2. Mismo proceso de verificación que los controles; 3. Exposición tasa de abandono.

### 3.1 Resumen de los Resultados

#### 3.1.1 Características Generales de la Revisión

El 54,54% de los artículos son del 2023 [25, 26, 27, 28, 29], el más antiguo data del 2022 [30] y los más nuevos del 2024 [24, 31, 33]. Por otra parte, esta revisión cuenta con un total de 1293 participantes evaluados longitudinalmente, de los cuales se reporta una pérdida de la muestra de 254 participantes. El 65,50% de los participantes de la muestra total estuvo compuesta por mujeres con una edad media de 46,24 años. En casi todos los estudios el porcentaje de mujeres incluidas fue muy superior al de los hombres, llegando incluso hasta el 78% de la muestra [32]. Solamente en dos estudios [30, 31] el porcentaje de hombres fue mayor al de mujeres, con un porcentaje del 54% y 59,25%, respectivamente. En uno de los anteriores estudios [30], con mayor proporción de hombres en la muestra se realizó una comparación entre géneros, encontrando que los hombres se vieron más afectados que las mujeres, al contrario que en el resto de los estudios.

#### 3.1.2 Pruebas Neuropsicológicas Utilizadas

Las pruebas neuropsicológicas utilizadas para medir la variable cognición resultaron muy heterogéneas. El Montreal Cognitive Assesment (MoCA) fue el test más utilizado [25, 28, 29, 32, 33], seguido del Trail Making Test (TMT) [25, 27, 31, 32], el test de Stroop [25, 31, 32], el Repeatable Battery for the Assessment of Neuropsychological Status (RBANS) [25, 27] y el Test of Premorbid Functioning (TOPF) [25, 32].

#### 3.1.3 Duración del Seguimiento

**Fig. 2.**
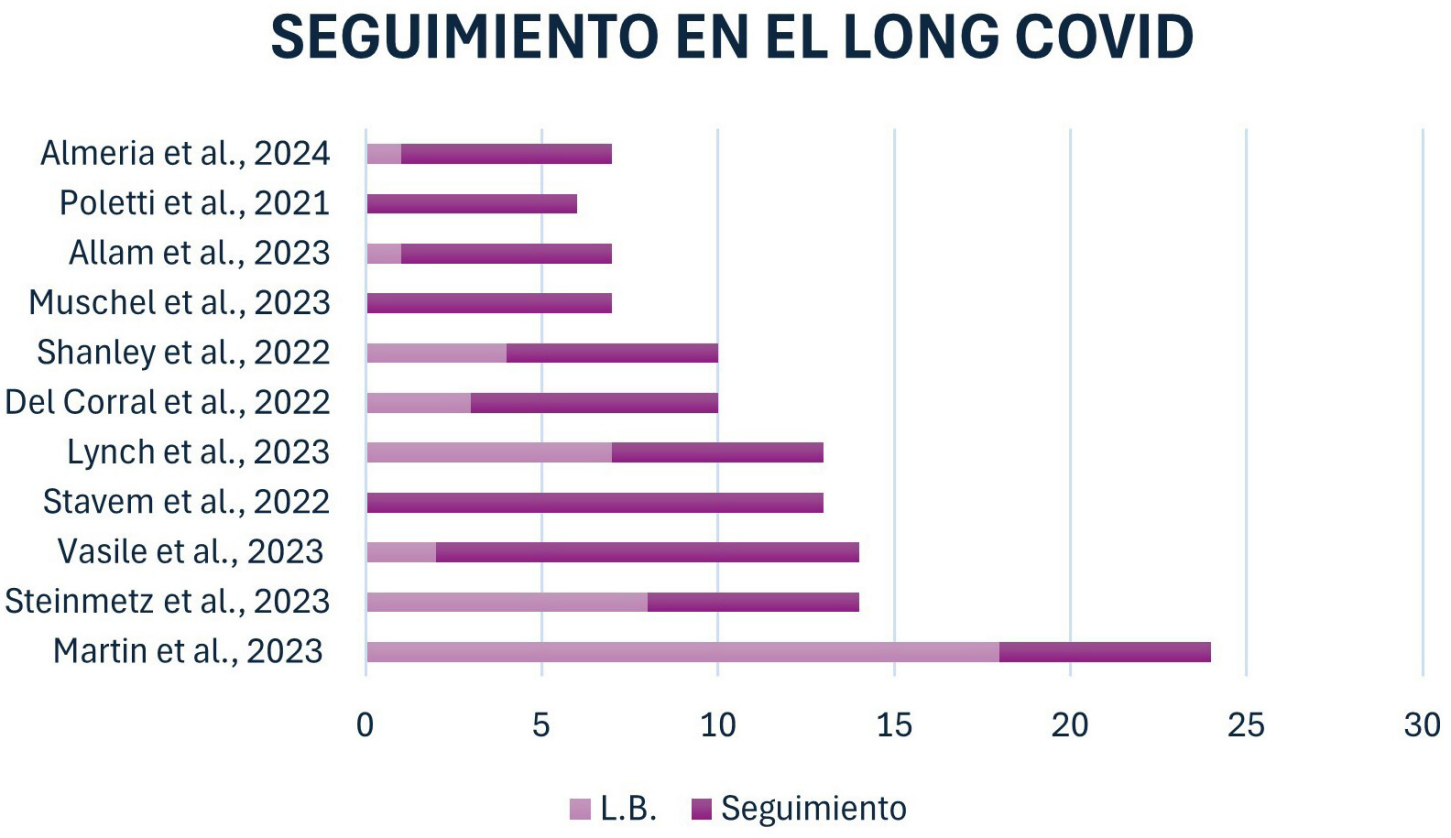
**Momento en el que se toma la línea base y número de meses de seguimiento de los estudios**. L.B., línea base.

La mayoría de los estudios siguieron a la muestra durante un tiempo que osciló entre los 6 meses y 1 año, aunque la mayor parte de los estudios realizaron un seguimiento de 6 meses [24, 26, 27, 28, 31, 32]. Sin embargo, como se observa en la Fig. 2, el tiempo en el que se tomaron medidas para la línea base (L.B.) se extiende desde un mes posterior a la infección por SARS-CoV, hasta incluso un año después, por lo que algunos estudios arrojaron resultados con respecto a la afectación en la cognición más de un año después [24, 28, 29, 34]. Por ello, a la hora de informar los resultados, se tuvo en cuenta el tiempo que pasó desde la infección. De esta manera, el rango temporal de los estudios que arrojaron resultados sobre la cognición abarcó desde los 18 meses tras la infección [24] hasta los seis meses más tarde [30, 31].

#### 3.1.4 Resultados Sobre el Tiempo que Permanecen Afectadas las Funciones Cognitivas

Los resultados de los estudios muestran que los problemas cognitivos en el *Long* COVID persisten a lo largo del tiempo y mejoran lentamente, aunque los estudios parecen coincidir en que la mayoría de las áreas mejoran significativamente a partir del año [24, 27, 28, 29].

#### 3.1.5 Resultados Acerca de Cuáles Son las Funciones Cognitivas más Afectadas

Existe mucha diferencia intragrupo, en función de donde proviene la muestra, respecto a cuáles son las funciones cognitivas que permanecen alteradas. Esto puede deberse a la variabilidad que hay entre los estudios con respecto de donde o cómo recogen la muestra o a la variedad de cuestionarios utilizados. Por lo que se observó en los estudios, la velocidad de procesamiento y la atención fueron las funciones cognitivas que más tiempo permanecieron afectadas [24, 27, 29, 33], seguido de la memoria (a corto o largo plazo, de trabajo) [27, 29, 33]. Respecto a las funciones ejecutivas, parece haber un acuerdo en su mejora al cabo de un año [24, 32, 33] a pesar de haber sido una de las funciones cognitivas más afectadas [25, 26, 30]. En cuanto al lenguaje, no se encontraron resultados confluentes respecto a su evolución. Algunos estudios no la nombraron como una de las áreas más afectadas al principio [25, 26, 30], pero sí a los 10 o 13 meses tras la infección [27, 32], y el estudio más largo encontró una mejora significativa a los 18 meses [24].

## 4. Discusión

Hasta lo que se sabe hasta la fecha, esta podría ser la primera revisión sistemática que analiza el impacto del *Long* COVID en la cognición, mediante estudios longitudinales en participantes de entre 18 a 65 años. Esta revisión cuenta, además, con profesionales capacitados que pasaron evaluaciones en su mayoría presenciales. Solo hubo un estudio [32] que dio la opción de evaluar presencial o telefónicamente, una vez tomada la línea base, para mejorar a la adherencia a la investigación. Estas características, pese a diferenciarse de otras revisiones, dificultaron la identificación de estudios elegibles.

Respecto a otras revisiones sistemáticas con objetivos similares, no se encontró ninguna investigación que excluyese estudios con participantes mayores a 65 años. Se sabe que uno de los principales factores de riesgo para desarrollar problemas cognitivos es la edad, por lo que dichas investigaciones contarían con un gran sesgo a la hora de dilucidar el impacto del COVID-19 sobre la cognición. Los problemas cognitivos identificados en dichas muestras podían deberse al inicio de un proceso de deterioro cognitivo de inicio previo a la infección. Esta limitación queda solventada en esta revisión al haber fijado una edad máxima de 65 años entre los participantes, edad a partir de la cual se ha encontrado que empiezan los problemas cognitivos asociados a la edad. A pesar de no compartir los mismos criterios de inclusión, nuestros hallazgos concuerdan con otras revisiones similares y al mismo tiempo quedan respaldados por estudios de neuroimagen.

En la revisión llevada a cabo por Perrottelli *et al*. [10] encontraron en estudios transversales y longitudinales de corta duración, que los dominios más afectados fueron las funciones ejecutivas, la atención, la velocidad de procesamiento y la memoria (visoespacial, episódica y de trabajo). Sin embargo, se incluyeron a participantes de hasta 96 años con evaluaciones no presenciales. Por otra parte, a diferencia de nuestra muestra, la suya estuvo compuesta principalmente por hombres que resultaron ser el género más afectado, al contrario de lo que se encontró aquí. Pese a que los estudios no demuestran una confluencia exacta respecto a que género es más vulnerable a padecer problemas cognitivos, algunos estudios recientes afirman que los hombres son más propensos a padecer problemas respiratorios y las mujeres problemas neuropsicológicos en el *Long *COVID. La mayor prevalencia de problemas cognitivos en las mujeres se debería a factores hormonales que propiciarían que estas tuvieran una mejor respuesta inmunoinflamatoria, que se relacionaría con una infección aguda menos grave que en los hombres, pero con propensión a desarrollar secuelas post-agudas del SARS-CoV-2 [3, 4]. En la misma línea, otras revisiones parecidas a la anterior, y cuyos estudios en su mayoría usaron el MoCA, también encontraron que las funciones cognitivas más afectadas fueron los dominios de la atención, la memoria y las funciones ejecutivas, sin embargo, no nombraron la velocidad de procesamiento [36, 37]. Ello se debe a que el MoCA no mide este dominio en particular.

Los estudios de neuroimagen publicados al respecto explicarían estas disfunciones cognitivas, al mostrar que el cerebro sufre una alteración en la organización funcional del conectoma [13, 38, 39]. En dichos estudios se encontró una hipoconectividad generalizada en pacientes con COVID-19. Concretamente, las regiones orbitofrontales y el giro parahipocampal fueron algunas de las zonas más afectadas. Estas regiones son especialmente importantes para el desarrollo de las funciones ejecutivas y la memoria, respectivamente. Por otra parte, tanto la disminución a gran escala de la conectividad cerebral como su reorganización explicarían la reducción de la eficiencia del procesamiento de la información encontrada en estos pacientes. De igual manera, en otro estudio de neuroimagen [40], hallaron que detrás de la disfunción cognitiva de estos pacientes también había niveles más altos de anisotropía fraccional de sustancia blanca, es decir daños axonales en sustancia blanca. Estos hallazgos explican la afectación en la velocidad de procesamiento y la atención que presentan este tipo de pacientes y, en conjunto, manifiestan la necesidad de una intervención cognitiva específica.

Respecto a la duración de problemas cognitivos en el *Long* COVID, los estudios hallados que lo evaluaban siguieron encontrando deterioro cognitivo en las muestras hasta 12 meses después [41, 42]. Incluso uno de los estudios longitudinales más largos hasta la fecha, detectó todavía deficiencias cognitivas dos años después de la infección [43].

Este trabajo, a pesar de ser una revisión que aporta nueva información con respecto a las secuelas cognitivas en el COVID-19, no está exento de limitaciones a partir de los estudios revisados. Hay una prevalencia de un sesgo de selección en las muestras. Algunos estudios componen sus muestras de programas de rehabilitación post-COVID [27, 28] o de pacientes que acuden al hospital por quejas subjetivas cognitivas [25, 26, 32]. Las personas que recibieron tratamiento previamente a las mediciones podrían tener un curso diferente en los déficits cognitivos presentados. Así mismo, la anosognosia cognitiva es común entre personas con deterioro cognitivo, por lo que las muestras de los estudios no representarían la totalidad de casos de deterior cognitivo en el *Long* COVID. Stavem *et al*. [34] decidieron no incluir en la muestra a participantes que hubiesen presentado disnea o fatiga, y según la literatura científica ambos síntomas están relacionados con el desarrollo de síntomas neuropsicológicos [31, 44, 45, 46]. En la misma línea, otro estudio solamente compuso su cohorte con pacientes no hospitalizados por COVID [33], por lo que la muestra deja de ser representativa.

Por otra parte, como se ha visto en los estudios incluidos, la población con *Long* COVID presenta altos niveles de ansiedad, estrés o incluso trastorno por estrés post traumático [24, 28]. Las variables emocionales podrían explicar una parte significativa de la varianza observada en el rendimiento de pruebas neuropsicológicas, y no todos los estudios incluyeron mediciones de las variables emocionales para controlar su efecto sobre la cognición [29]. En uno de los estudios incluidos que sí controlaron estas variables emocionales [31], encontraron que el grupo con *Long* COVID que menos mejoría presentó en la función cognitiva fue el grupo con mayores puntuaciones en ansiedad y depresión, por lo que esta sintomatología podría influir en la recuperación cognitiva.

Por otro lado, en cuanto a las baterías neuropsicológicas utilizadas, no todas fueron igual de sensibles para captar el deterior cognitivo leve. Se ha demostrado que el MoCA es más sensible en captar el deterioro cognitivo que el Mini-Mental State Examination (MMSE) [47]. En la misma línea, la heterogeneidad de las pruebas utilizadas ha permitido ver que no todas miden el mismo constructo (cognición) de igual forma.

En conclusión, en la línea de todo lo comentado anteriormente con respecto a la diversidad de participantes incluidos en las muestras, así como la heterogeneidad de las pruebas utilizadas, y los diferentes momentos temporales en los que se toma la línea base dificulta poder sacar una conclusión respecto a los dos objetivos marcados de la revisión. Estas limitaciones se deben a la falta del establecimiento de un protocolo que guíe la investigación del impacto de *Long* COVID en la cognición. Se recomienda el encuentro entre un grupo de expertos de la Sociedad Española de Neurología (SEN) y de la Federación de Sociedades de Neuropsicología Españolas (FANPSE) para llegar a un consenso, como en otras disciplinas o patologías se ha hecho anteriormente, respecto a que protocolo de investigación se debería implementar para que futuras investigaciones pudiesen tratar este tema con el menor sesgo posible.

## Data Availability

Los datos ya se proporcionan en el artículo.

## Contribuciones de los Autores

MATP y RET diseñaron el estudio de investigación. MATP realizó la investigación. MGD analizó los datos del estudio. MATP redactó el manuscrito y RET y MGD corrigieron y mejoraron el original. Todos los autores leyeron y aprobaron el manuscrito final. Todos los autores han participado lo suficiente en el trabajo y han acordado ser responsables de todos los aspectos del mismo.

## Aprobación Ética y Consentimiento Informado

No aplicable.

## Financiación

Esta investigación no recibió ningún tipo de financiación externa.

## Material Suplementario

El material suplementario asociado con este artículo se puede encontrar, en la versión en línea, en https://doi.org/10.31083/RN37385.
